# Mesenchymal Stem Cells with Granulocyte Colony-Stimulating Factor Reduce Stress Oxidative Factors in Parkinson's Disease

**DOI:** 10.29252/ibj.24.2.89

**Published:** 2019-11-02

**Authors:** Laya Ghahari, Manouchehr Safari, Khojaste Rahimi Jaberi, Behnaz Jafari, Katayoun Safari, Mahmoodreza Madadian

**Affiliations:** 1 Department of Anatomy, AJA University of Medical Sciences, Tehran, Iran;; 2 Nervous System Stem Cells Research Center, Semnan university of Medical Sciences, Semnan, Iran;; 3 Shahrood Medical University, Shahrood, Iran;; 4 Faculty of Veterinary Medicine, University of Tehran, Tehran, Iran;; 5 School of Pharmacology, Pharmaceutical Sciences Branch, Islamic Azad University, Tehran, Iran

**Keywords:** Granulocyte colony-stimulating factor, Mesenchymal stem cells, Parkinson disease, Oxidative stress

## Abstract

**Background:**

Recent studies have shown that BMSCs have a putative ability to promote neurogenesis and produce behavioral and functional improvement. Our previous study demonstrated that co-treatment of G-CSF and BMSCs have beneficial effects on Parkinson's models. The main purpose of this research was to investigate the effects of these two factors on oxidative stress factors in the brain of Parkinson's rat.

**Methods:**

Adult male Wistar rats (weighing 200–250 g) were used and randomly divided into five groups of seven each. To create the Parkinson's model, 6-OHDA was injected into the left SNpc. The BMSCs (2 × 10^6^) and G-CSF (75 µg/kg) were used for treatment after creating the PD model. After four weeks, the brains of rats were removed and processed for immunohistochemical studies, such as TH-positive neurons as well as analysis of oxidative stress factors.

**Results:**

The results showed that the injected BMSCs could cross the BBB. The injected cells are also able to settle in different areas of the brain. Analyses of the brain oxidative stress factors showed that G-CSF and BMSCs reduced the expression of MDA and induced the activity of SOD, GSH-Px, and FRAP.

**Conclusion:**

Co-administration of G-CSF and BMSCs reduced the expression of pro-inflammatory cytokines and induced the activity of antioxidant enzymes; however, neurogenesis increased in the brain.

## INTRODUCTION

Parkinson's disease is a widespread and popular neurodegenerative disorder worldwide^[^^1^^]^. The main cause of this disease is the destruction of DA neurons in the pathway of nigrostriatal system. The symptoms of PD begin to appear when nearly 60% of the DA neurons of the SNpc is eliminated^[^^2^^]^. Different factors are involved in the development of PD. Oxidative stress is one of most the key factors affecting PD.

BMSCs are capable of renovating themselves. They can differentiate into other cells, depending on their fetal origin^[^^3^^]^. BMSCs express unique surface markers such as CD29 and CD44, but they are not able to express hematopoietic cell markers, CD34 and CD45^[^^4^^]^. One of the advantages of BMSCs, in contrast to embryonic stem cells, is the low possibility of rejection after transplantation. Hence, the application of adult BMSCs rather than embryonic stem cells has been recommended^[^^5^^]^. 

G-CSF is a growth factor with the molecular weight of 19.6 kDa. Some cells such as monocytes, macrophages, endothelial cells, and fibroblasts secrete G-CSF in the body. By inhibiting apoptosis process, G-CSF is able to increase the survival and proliferation of neutrophil. Receptors of G-CSF have been indicated to be able to re-express extensively in the CNS^[^^6^^]^. G-CSF can pass the BBB^[^^3^^]^, and in addition to the anti-apoptotic effect on the hematopoietic system, it has a neuroprotective impact on the 6-OHDA model of PD^[^^7^^]^ and brain damage after hypoxia^[^^8^^]^. This neuroprotective effect is mediated by direct activity function of G-CSF on neurons. G-CSF has also strong anti-apoptotic activity in neuronal cells^[^^9^^,^^10^^]^ and induces the pathways of STAT3 and ERK 1/2/5 to promote the survival of the CNS^[^^9^^]^. However, in other cell types, G-CSF activates ERK5 pathway^[^^11^^]^. The greatest effect of the anti-apoptotic activity of G-CSF is in the PI3K/Akt pathway^[^^12^^]^. G-CSF induces neurogenesis in the CNS^[^^13^^]^ and decreases systemic inflammatory reactions^[^^14^^]^.

Research has confirmed the key role of oxidative stress in PD pathogenesis. For instance, it has been shown that the increased level of oxidative stress markers in the SNpc induces nigral cell degeneration^[^^15^^]^. Oxidative stress enzymes such as SOD, CAT, and GSH-Px act as causative factors in promotion of several neurodegenerative disorders, such as PD, Alzheimer, and amyotrophic lateral sclerosis^[^^16^^]^. Oxidative stress can initiate the mitochondria and endoplasmic reticulum dysfunction, leading to protein misfolding in neurons and then apoptosis. Mitochondria are important organells in the production of ROS in the cells^[^^17^^]^. Dysfunction of mitochondria results in decreased ATP production. The accumulation of oxidative stress causes changes in mitochondria, including deformation, calcium channel destruction, DNA damage, and eventually cell death^[^^18^^]^. Destruction of mitochondrial complex I is the main cause of ROS accumulation in DA neurons in PD, and following the destruction, the respiratory electron transport chain will be arrested, and the production of oxygen decreases, which in turn result in elevated levels of ROS products inside the cells^[^^19^^]^. Therefore, the irregular activity of mitochondria is the main source of intracellular ROS production that will lead to the destruction of DA neurons in PD^[^^20^^]^. The main purpose of this research was to investigate the effects of mesenchymal stem cells and G-CSF on oxidative stress in the brain of Parkinson's rat.

## MATERIALS AND METHODS

**Experimental protocol**

A total of 35 adult male Wistar rats, weighing 200-250 g at the beginning of the experiment, were provided by the Experimental Center of Semnan Medical University, Semnan, Iran. All the rats were housed at three or four rats per cage with free access to food and water. The temperature of the storage room was maintained at 20–23 °C and simulated daylight conditions (12-h dark and 12-h light). All the stages of testing were carried out in accordance with the Ethics Committee of Semnan Medical University Semnan (ethical no. 677). Rats were randomly divided into five groups (n = 7 in each group). The first group received only culture media (DMEM) as the control. The second group was given 4 µg of 6-OHDA in the left substantia nigra as the PD model. The third group received BMSCs (2 × 10^6^) by dorsal caudal vein one week after PD. The fourth group was treated with G-CSF (75 µg/kg) intraperitoneally for seven days, one week after PD. The fifth group received BMSCs (2 × 10^6^) by dorsal caudal vein and G-CSF (75 µg/kg) intraperitoneally for seven days, one week after PD. All the rats were decapitated four weeks after the last behavioral test, and their brains were prepared for immunohistochemical investigations, including staining of TH-positive cells and analysis of stress oxidative markers.

**The 6-OHDA** **lesion**

For the unilateral destruction of the nigral system, 6-OHDA was injected into the left SNpc. For anesthesia, the intraperitoneal injections of ketamine hydrochloride and xylazine hydrochloride (100 mg/kg and 20 mg/kg, respectively; Sigma-Aldrich, USA) were used. Rats were then placed in a stereotaxic instrument (Stoelting, USA). The skull was exposed by an incision in the scalp, and a single hole was drilled on the left side of the SNpc at the following positions according to the rat brain in stereotaxic coordinates: AP = -4.8 mm anterior to bregma, ML = -1.6 mm lateral to the midline, DV = 8.2 mm vertical from the dura. Next, 4 µl of 6-OHDA (2 µg/µl) dissolved in 2 mg/ml of ascorbic acid solution (Sigma-Aldrich) in saline was administrated to the SNpc with a 28-gauge Hamilton syringe. Next, 6-OHDA was injected to the left side at the rate of 1 µl/min^[^^21^^]^. 

**Behavioral testing**

Motor imbalance was evaluated by the apomorphine-induced rotational test. All behavioral testings were performed by an observer blinded to the group. All the groups were tested for rotational behavior one week after the first surgery and also 1, 2, 3, 4 weeks after the treatment. The apomorphine hydrochloride of 2.5 mg/kg (Sigma-Aldrich) was used for the behavioral test. Complete rotations in the opposite direction of the lesion were counted for 30 minutes. The value was expressed as contralateral net turns/min.

**BMSCs** **culture** ***in vitro***

BMSCs were isolated under the sterile conditions from tibias and femurs of adult male Wistar rats (n = 7; 220- 250 g). The bone marrow was flushed with Hank's balanced salt solution using a syringe with a 21-gauge needle. For removing debris, the cell suspension was filtered through a cell strainer (100 μm), and then all the cells were centrifuged and cultured into each 75-cm^2 ^culture flask containing Dulbecco's modified eagle medium (Invitrogen, UK) supplemented with 10% fetal bovine serum (Gibco, Dublin, Ireland), 1% (v/v) penicillin/streptomycin (Gibco) in a 5% CO_2_ incubator at 37 °C. After two days, non-adherent cells were decanted, and adherent cells were used. The BMSCs were isolated on the basis of their ability to adhere to the flasks. Subsequently, incubation was continued, and the medium was changed at three-day intervals. BMSCs were allowed to grow until 70–80% confluency and then subjected to flow cytometry analysis.

**Analysis of cell surface antigen markers**

The expression of surface markers on mesenchymal stem cells was performed after the third cell passage by flow cytometry technique. Briefly, cells from bone marrow source were harvested and centrifuged at 180 ×g at 4 °C for 3 min. The pellets were washed three times with a cold stain buffer, filtered through a cell strainer (100 µm) and re-suspended in cold stain buffer to the concentration of 2 × 10^4^ cells/ml. Here, a combination of positive and negative markers were used. In this regard, CD34 and CD45 (as hematopoietic markers) and CD29 and CD44 (as mesenchymal markers) were considered to characterize the isolated BMSCs. Cells were incubated directly with fluorescence-labeled monoclonal antibodies against CD29, CD34, CD44, and CD45 (Sigma Aldrich). Samples were analyzed using a FACSCalibur flow cytometry apparatus^[^^22^^]^.

**DiI labeling**

CM-DiI is a carbocyanine membrane dye that exhibits increased fluorescence upon the insertion of its lipophilic hydrocarbon chains into the lipid membrane of cells. The high photostability and continual fluorescence of the dye serve as an effective dye for the recognition of neuronal structure. Fluorescence of CM-DiI-labeled BMSCs showed a strong red signal at 600 nm. For CM-DiI labeling of BMSCs, these cells were incubated with 5 µg CM-DiI /10^6^ cells (Molecular Probes, Invitrogen, USA) under 5% CO_2_ at 37 °C for 2 h. Then 2 × 10^6 ^cells were separated and injected into the vena caudal.

**Immunohistochemical and histological study**

In order to investigate DA neuronal population within the SNpc, immunohistochemical study was conducted. Ater anesthesia, animals were intracardially perfused with saline and 4% fixative solution of paraformaldehyde in 0.1 M of phosphate buffer (pH 7.4). The brains were removed from the skull and fixed in 2.5% paraformaldehyde for one week. Coronal sections of 6 µm were then prepared. Tissue sections were blocked in 10% methanol and H_2_O_2 _at darkness for 8 min, washed several times with Tris (pH 7.4) and incubated in the citrate buffer (pH 7.6) at 98 °C for 10-15 min. The sections were then washed three times (each time for three min) with Tris (pH 7.4) and then blocked in 10% normal goat serum, 1% BSA, 0.3% Triton X-100 in PBS at 25 °C for 2 hours. Sections with the primary antibody (1:500; Abcam, Germany) were incubated with 0.3% TBS/1% BSA at 4 °C overnight. The sections were then washed three times in TBS for 5 min. Biotin-conjugated secondary antibody incubation (1:100; Abcam) was performed at room temperature (25 °C) for 2 h. After several washes in PBS, cells were incubated at 25 °C in 1% 3-3’-diaminobenzidine (Abcam) at darkness for 10 minutes. The sections were then mounted onto gelatin-coated slides and coverslipped after dehydration in ascending concentrations of ethanol-xylene solutions. The Dil-labeled BMSCs were prepared as described above and injected into the dorsal caudal vein of rats. The numbers of stained cell bodies were counted by an observer blinded to the history of treatment. NIH Image J software was used for counting the sections. After determining the area of the SNpc at low magnification (objective 4×) to avoid the repeated counting of neurons, immunopositive cells were counted only when their nuclei were optically visible. 

**Measurement of oxidative stress markers**

At the end of the experiments, after deep anesthesia, rats were decapitated, brains were removed and dissected on an ice-cold glass plate. For assessing stress oxidative markers such as MDA, SOD, GSH-Px, and FRAP, after decapitation, the bilateral midbrain immediately was isolated from the brain stems. Samples were weighed exactly and prepared with 0.90% normal saline to give 10% tissue homogenate by super-audible cell disintegrator (Sonicater), then centrifuged at 3000 ×g/min at 4 °C for 20 min. The supernatant was collected and kept at -70 °C until use. The protein concentration of the substantia nigra was determined by Bradford method^[^^23^^]^. The activities of MDA, SOD, and GSH-Px, and the content of FRAP in the midbrain were determined following the kit specifications (Abcam).

**Statistical analysis**

Results were presented as the units of activity per mg of protein (wet weight) or content. All data were expressed as mean ± SD. Data were analyzed by one-way ANOVA, followed by Tukey's post hoc test for DA neuron counts. A value of *p *< 0.05 was considered to be statistically significant.

## RESULTS

**Flow cytometry**

**Fig. 1:**
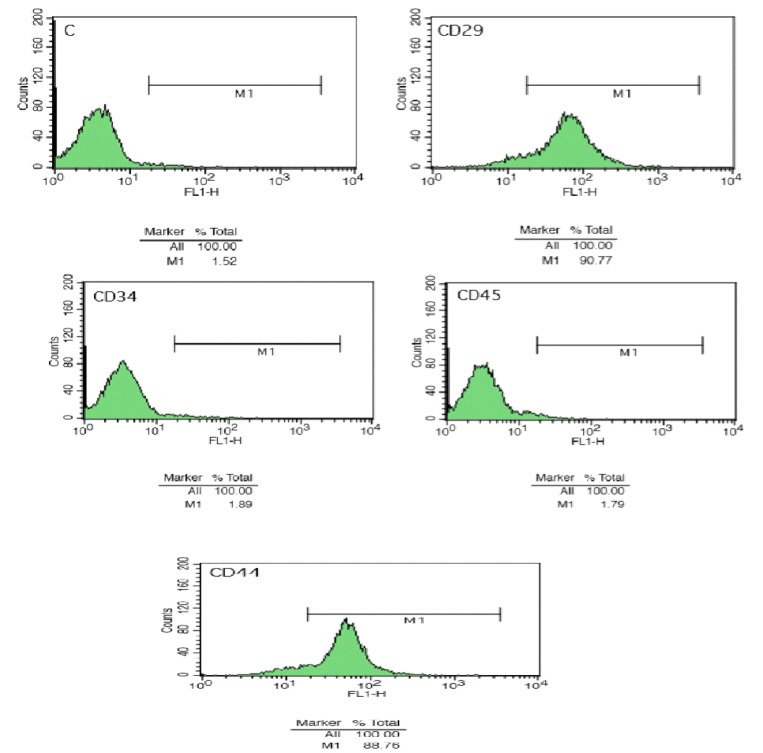
Detection of cultured cells by flow cytometry. Flow cytometry showed that the BMSC were negative for surface expression of CD45 and CD34 and positive for surface expression of CD29 and CD44. C, control

Flow cytometry analysis of BMSCs after the third passage demonstrated that the cells were negative for surface expression of CD45 and CD34, but positive for surface expression of CD29 and CD 44, indicating the presence of stem cell markers on the surface of isolated cells (Fig. 1).

**Behavioral results**

**Fig. 2:**
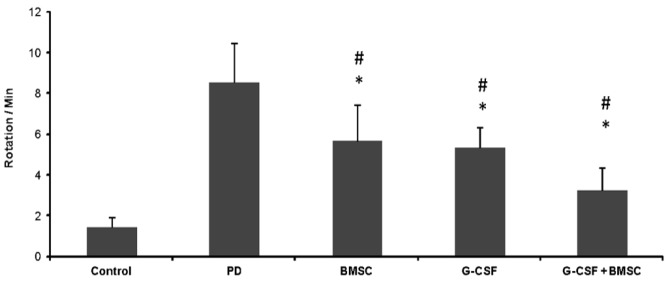
Effects of BMSCs and G-CSF on behavioral tests. Apomorphine-induced rotations of rats over time revealed a significantly decreased number of rotations in G-CSF plus BMSCs treatment group. In all treatment groups, rotations significantly decreased, but no significant difference was observed between BMSC and G-CSF. There were statistically significant between G-CSF plus BMSCs and G-CSF as well as BMSCs. All values were mean ± SD. ^*^compared with the PD group (*p* ˂ 0.05), ^#^compared with the control (*p* ˂ 0.05)

The opposite rotation was induced by apomorphine hydrochloride in rats inside the cage (Fig. 2). In the PD group, contralateral rotation was higher and significant compared to the control group (*p* < 0.01). The mean rotation in the PD group was 8.56 ± 2.1 circles/min. Rotation in the PD group was also higher than that of all the treatment groups, and the difference was significant, as compared to the other groups (*p* < 0.01). There was no significant difference in the number of rotations in BMSCs therapy and G-CSF (*p* < 0.01). In the group of G-CSF plus BMSCs, contralateral rotations significantly ameliorated in comparison to the other groups (G-CSF plus BMSCs: 4.25 ± 1.2, G-CSF: 5.33 ± 1.4, BMSCs: 5.66 ± 1.7, and control : 1.46 ± 0.1; *p* < 0.01). In all the groups, the mean rotations at the end of tests were less than those at the beginning. In the G-CSF plus BMSC groups, the number of rotations at the end of treatment was lower than all other treatment groups and was close to the control group.

**Immunohistochemistry**

**Fig. 3:**
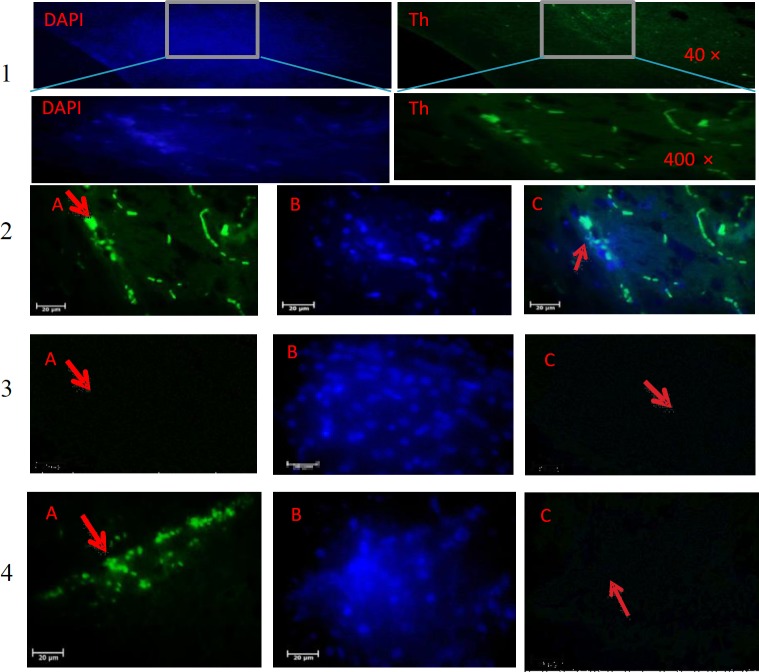
TH immunohistochemistry staining of the substantia nigra of the rat. (1) control, (2) BMSCs, (3) G-CSF, and (4) G-CSF plus BMSCs groups. Arrows show TH-positive neurons. The number of TH-positive cells significantly increased after treatment with G-CSF plus BMSCs compared to the control groups. There were not statistically significant in BMSCs and G-CSF in comparison with the PD group. (A) primary antibody to TH, (B) nuclei stained by DAPI, and (C) the merged picture of A and B

**Fig. 4:**
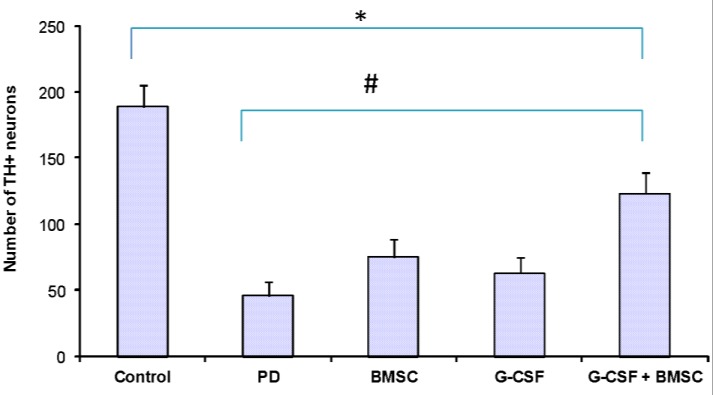
Effects of BMSCs and G-CSF on the number of TH-positive cells. The Figure also shows the mean number of TH-positive neurons in the SNpc of all groups. The number of TH-positive neurons in the SNpc were significantly higher in the group received  G-CSF plus BMSCs. ^*^compared with the control group (*p* ˂ 0.05), ^#^compared with the PD group (*p* ˂ 0.05). Values were mean ± SD

TH staining revealed the significant reduction of TH^+^ cells in the 6-OHDA group in SNpc (46.2 ± 3.2, *p* < 0.001; Figs. 3 and 4). As shown in Figure 4, in the control group, the number of TH- positive neurons was 189.1± 9.5 (*p* < 0.001), and there were significant differences between the control and PD groups. The TH^+ ^neurons in the G-CSF and BMSCs groups were 63.2 ± 7.2 and 75.3 ± 4.9, respectively, but 123.5 ± 7.3 in the G-CSF plus BMSCs group (*p* < 0.05). The highest number of TH^+ ^neurons was observed in the G-CSF plus BMSCs group. There was a significant difference between the PD and other therapeutic groups (*p* < 0.001). The results confirmed significant difference between the G-CSF and BMSC groups (*p* < 0.05). TH staining showed significant preservation of DA neurons in all treatment groups. Therefore, it seems that G-CSF and BMSCs protect DA neurons in SNpc or may cause the migration and differentiation of stem cells from other areas of the affected site.

**CM-DiI staining**

**Fig. 5:**
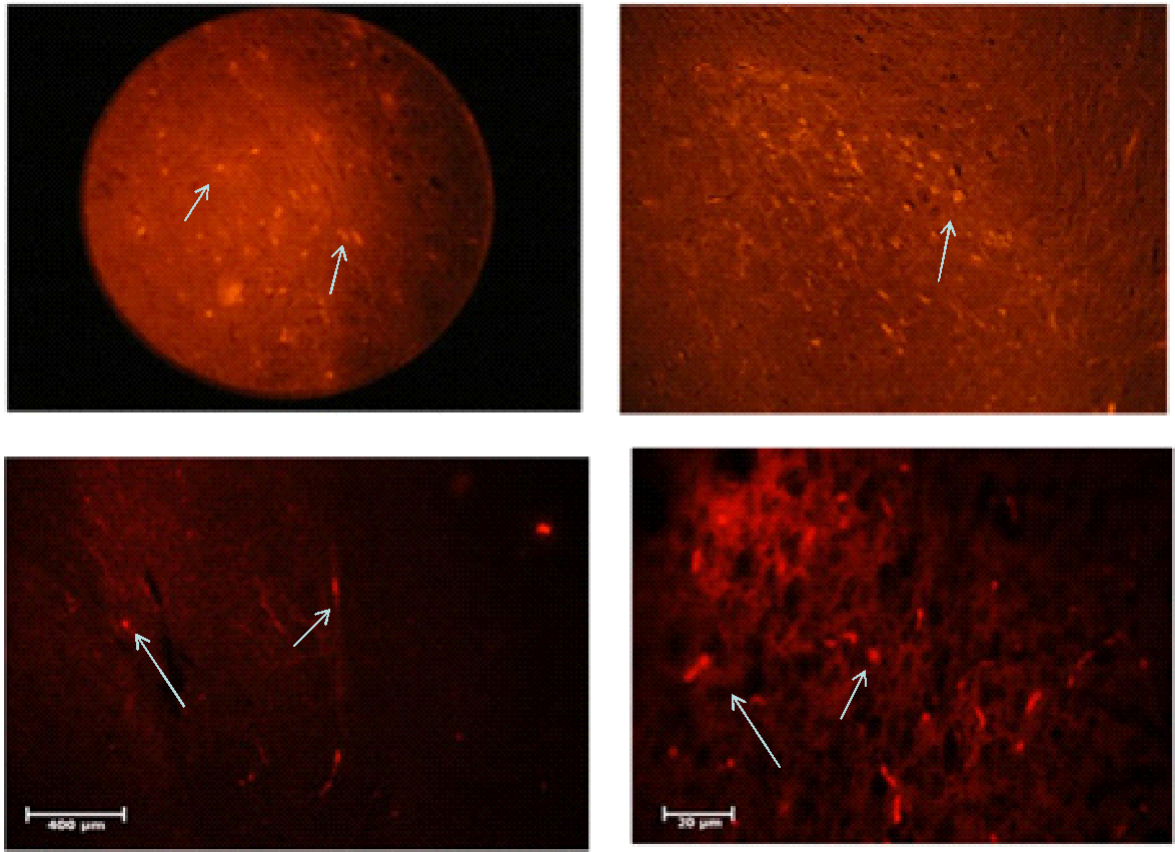
Location of DiI-stained cells. DiI Staining revealed dispersion of injected cells in the different parts of the brain and a lot of cells in the SNpc. The arrows indicate MTT-stained cells entering different parts of the brain

CM-DiI staining was carried out to assess the location of transplanted cells. In the histological sections, it was found that the injected cells were distributed sporadically in different parts of the brain, and a large numbers of cells are located in the SNpc (Fig. 5).

**Oxidative stress markers**

The results of oxidative stress tests showed changes in lipid peroxidation and antioxidant enzymes after the administration of BMSCs and G-CSF (Table 1). MDA levels significantly increased in the 6-OHDA (PD group) and all treated groups when compared to the control group. The level of MDA in the PD group was almost twice as much as the control group (6.70 ± 0.22 vs. 3.10 ± 0.22). MDA levels significantly decreased following the administration of G-CSF alone (19.8%) and in combination with BMSCs (22.4%). In comparison to PD, the maximum decrease was observed in the G-CSF plus BMSCs group (5.20 ± 0.16). MDA levels between the BMSCs and the PD groups were not significant (6.44 ± 0.11 vs. 6.70 ± 0.22; *p* ≤ 0.01). The levels of GSH-Px significantly decreased following treatment with 6-OHDA as compared with the control (3.12 ± 0.15 vs. 6.23 ± 0.12). GSH-Px amount in animals co-administrated with G-CSF plus BMSCs (5.16 ± 0.11) and G-CSF alone (4.69 ± 0.11) was closer to the control group (6.23 ± 0.12). There were no significant differences between BMSCs treatment and the PD group (4.14 ± 0.08 vs. 3.12 ± 0.15). Also, no significant difference was found between G-CSF plus BMSCs and the control groups (5.16 ± 0.11 vs. 6.23 ± 0.12). Based on the the data in Table 1, there was a significant decrease in the level and activity of SOD by 6-OHDA treatment compared to the control (10.2 ± 3.17 vs. 23.21 ± 2.19; *p* ≤ 0.05). The activity of SOD in PD group reduced by 44% when compared to the controls. However, in G-CSF and G-CSF plus BMSCs receiving groups (14.26 ± 3.11 and 17.66 ± 0.11, respectively), the activity increased compared to the control (23.21 ± 2.19). Also, there were no significant differences between the BMSCs-treated group and PD group (11.35 ± 2.11 vs. 10.2 ± 3.17). Results showed a significant decrease in the activity of FRAP upon 6-OHDA treatment compared to the control (0.52 ± 0.09 vs. 2.79 ± 1.15; *p* ≤ 0.05). FRAP level significantly increased in the G-CSF plus BMSCs (1.79 ± 0.3) and G-CSF administration alone 1.13 ± 0.1), but in the BMSCs-treated group, no significant change was found in comparison to 6-OHDA group.

## DISCUSSION

The results of immunohohechemical staining showed that G-CSF is capable of migrating injected stem cells into the SNpc. It also changed the stress oxidative factors in the brain. The G-CSF is one of the most important components of the hematopoietic growth factor family^[^^23^^]^. Studies have shown that in addition to neuroprotective effect, G-CSF has a potential role in the migration of progenitor cells from bone marrow to peripheral blood^[^^24^^]^. The neuroprotective effect of G-CSF has been attributed to the inhibitory activity of G-CSF on the main inflammatory mediators such as interleukin-1, tumor necrosis factor-alpha, and interferon gamma^[^^9^^]^. More importantly, G-CSF is able to cross the BBB and induces neurogenesis in the CNS, increases synaptic plasticity and influences neurons and glial cells in the injured area. G-CSF has also inhibitory actions upon pro-inflammatory cytokines while up-regulating neurogenesis^[^^25^^]^. Similar to G-CSF, stem cells are able to cross the BBB. G-CSF causes the migration of stem cells from the bone marrow to the bloodstream. Therefore, G-CSF is capable of migrating injected stem cells from the blood into the brain^[^^5^^]^.

In this study, 2 × 10^6^ BMSCs were injected via the dorsal caudal vein. This number of BMSCs, which was chosen to prevent clotting in the bloodstream, is usually used for *in situ* injection in the brain. The dorsal caudal vein was selected to prevent any damage to the brain.  Exogenous cells may be trapped in the shown that parenchyma of some internal organs such as the lungs, liver, and even in the spleen. Our previous study has the low number of injected cells (2 × 10^5^ BMSCs) from the dorsal caudal vein with G-CSF (70 µg/kg) could not promote the migration of BMSCs into the brain^[^^1^^]^. In this project, after DiI labeling, we observed that, the labeled BMSCs were present in different parts of the brain and even in SNpc. We concluded that G-CSF facilitates the transit of the stem cells from the BBB and deposits into different parts of the brain. In addition, the injection of the BMSCs or G-CSF alone led to the behavioral improvement on animals, but no significant difference was observed between these two treatment groups. On the other hand, the number of TH^+ ^neurons between the two groups did not show any significant difference. Therefore, we draw the conclusion that the injection of BMSCs or G-CSF (75 µg/kg) gives similar results. Treatment with the optimal dose of G-CSF (75 µg/kg) combined with BMSCs significantly increased TH^+ ^neurons in comparison to all the treatment groups. Therefore, these two factors seem to have a synergistic effect. BMSCs will protect the DA neurons by modulating the activity of microglial cells as well as modulating their anti-inflammatory effects^[^^26^^]^. We suppose that BMSCs and G-CSFs can trigger certain factors or modulate expression of pro-inflammatory cytokines, which have a protective effect on DA neurons. The results showed statistically significant differences between the group of G-CSF plus BMSCs and BMSCs and G-CSF alone. It can be concluded that G-CSF is cooperated synergistically with BMSC. The co-administration of G-CSF and BMSCs facilitates the stability of the damaged area. G-CSF plays a role in healing the damaged nervous system by activating mechanisms such as anti-inflammatory pathways^[^^27^^]^, anti-apoptotic effects, differentiation of adult stem cells, induction of angiogenesis and vasculogenesis, and renovation of the BBB^[^^28^^]^ .

**Table 1:** Effect of BMSC and G-CSF on the level of stress oxidative factors

**Groups (n = 5)**	**GSH-Px (IU/ml)**	**SOD (IU/ml)**	**MDA (nmol/ml)**	**FRAP(µmol/ml)**
PD	3.12 ± 0.15	10.2 ± 3.17	6.70 ± 0.22	0.52 ± 0.09
Control	6.23 ± 0.12*	23.21 ± 2.19*	3.10 ± 0.22*	2.79 ± 1.15*
G-CSF	4.69 ± 0.11*#	14.26 ± 3.11#	5.38 ± 0.24*#	1.13 ± 0.1*#
BMSCs	4.14 ± 0.08#	11.35 ± 2.11#	6.44 ± 0.11#	0.73 ± 0.12#
G-CSF + BMSCs	5.16 ± 0.11*	17.66 ± 0.11*#	5.20 ± 0.16*#	1.79 ± 0.3*#

The levels of SOD and GSH-Px significantly increased and MDA decreased after treatment with G-CSF plus BMSCs in comparison to PD. There was not statistically significant difference in all enzymes in BMSCs group compared with PD groups. Values were mean ± SD. ^*^compared with the 6-OHDA (PD) group (*p* ˂ 0.05). ^#^compared with the control group (*p* ˂ 0.05).

The results from our study revealed that the level of MDA increased in the brain of PD group, thereby increasing the lipid peroxidation in PD rats. Our results also showed a decrease in the level of MDA in G-CSF and G-CSF plus BMSCs groups. These results are consistent with previous studies that reported enhanced oxidative stress markers in the plasma, cerebrospinal ﬂuid, and the substantia nigra in PD^[^^29^^]^. We assume that G-CSF is the specific factor for the reduction of MDA and acts as an antioxidant. Therefore, it is likely that MDA levels are reduced by G-CSF through the boosting of release, migration and differentiation of BMSC, which ultimately results in the protection of damaged cells. Our investigations demonstrated that G-CSF significantly increased the level of SOD and GSH-Px compared to the PD group. The results also showed that the activity of the GSH-Px decreased in PD patients, but significantly increased in the G-CSF and G-CSF plus BMSC groups compared to the PD group. These results are in agreement with that of other studies showing that GSH-Px activity decreased in the substantia nigra of PD patients^[^^30^^]^. 

In Parkinson’s patients, reduction of erythrocyte GSH-Px activity could lead to the accumulation of H_2_O_2_ in the SNpc. This part of the brain contains a high concentration of iron. All the conditions are gathered together for the transformation of H_2_O_2_ into OH by the Fenton’s reaction^[^^31^^]^. This free active radical can attack and destruct both lipids and proteins and then leads to the neuronal oxidative damage. In the brain, H_2_O_2_ is mainly catabolized by GSH-Px, a major enzyme in the development of neurodegenerative diseases^[^^32^^]^. Our study disclosed that G-CSF could result in the elevation of GSH-Px in SNpc. The key roles of G-CSF in CNS have been identified in new studies. G-CSF is able to bind to specific receptors (G-CSF receptor) in different cells such as monocytes, hematopoietic progenitor cells, neurons, platelets, endothelial cells, and small-cell lung cancer cells^[^^33^^,^^34^^]^. With the activation of the G-CSF receptors, the signaling cascades including the Janus kinase/signal transducer and transcription activator, Ras/mitogen-activated protein kinase, and phosphatidylinositol 3-kinase/Protein kinase B/Akt pathways will be stimulated. Activation of these cascades leads to the cellular proliferation, activation of anti-inflammatory and antiapoptotic processes and also mobilizes stem cells to target sites (sites of injury)^[^^14^^]^. According to these findings, G-CSF is a new, attractive and cost-effective factor for the treatment of neurodegenerative diseases. 

The present data indicate that G-CSF plays more important role than the BMSCs in PD. Increased MDA and decreased SOD and GSH-Px cause mitochondrial and then neuronal destruction. These events could eventually participate in PD pahogenesis. The decreased activity of these enzymes is indirectly responsible for neuronal loss and probably has an essential role in the onset of PD. The balance of these enzymes will protect nerve cells. Based on our findings, G-CSF administration along with BMSCs injection elevates SOD and GSH-Px in PD; however, extensive research is needed to further understand the mechanisms involved in the development of this disease.
